# Spatially- and vector-resolved momentum flux lost to a wall in a magnetic nozzle rf plasma thruster

**DOI:** 10.1038/s41598-020-58022-6

**Published:** 2020-01-23

**Authors:** Kazunori Takahashi, Takeharu Sugawara, Akira Ando

**Affiliations:** Department of Electrical Engineering, Tohoku University, Sendai, 980-8579 Japan

**Keywords:** Plasma physics, Fluid dynamics

## Abstract

Most of the artificial low-pressure plasmas contact with physical walls in laboratories; the plasma loss at the wall significantly affects the plasma device performance, e.g., an electric propulsion device. Near the surface of the wall, ions are spontaneously accelerated by a sheath and deliver their momentum and energy to the wall, while most of the electrons are reflected there. The momentum flux of the ions is a vector field, i.e., having both the radial and axial components even if the azimuthal components are neglected in a cylindrical system. Here the spatially- and vector-resolved measurement of the momentum flux near the cylindrical source wall of a magnetic nozzle radiofrequency (rf) plasma thruster configuration is successfully demonstrated by using a momentum vector measurement instrument. The results experimentally identify the spatial profile of a non-negligible axial momentum flux to the wall, while the radially accelerated ions seem to be responsible for the energy loss to the wall. The spatial profiles of the radial and axial momentum fluxes and the energy lost to the wall are significantly affected by the magnetic field strength. The results contribute to understand how and where the momentum and energy in the artificial plasma devices are lost, in addition to the presently tested thruster.

## Introduction

The plasma momentum is one of the fundamental physical quantities associated with static and dynamic phenomena of plasmas in nature and laboratories. The energy flux being a scalar quantity can be given by the magnitude of the velocity, while the momentum flux is a vector quantity in general. It is crucial to understand and characterize the momentum transport, conversion, gain, and loss mechanisms for clarifying the structural formation of plasmas such as the astrophysical jets^1^, the particle acceleration in auroras at the Earth and Jupiter^2,3^, the coronal mass ejection from the Sun^4^, the interaction between the plasmas and the geomagnetic field^5^, and so on. In these naturally appearing plasmas, the processes involving the momentum and energy transfer occur due to the interaction with electromagnetic fields, collisions, turbulences, and so on, since they cannot see any physical boundaries in space except for the surface of planets.

Terrestrial artificial plasmas ubiquitously contact to physical boundaries; forming a sheath maintaining charge balance in plasmas^6^. Plasma-wall interactions involving the momentum transfer and loss also significantly affect the plasma behavior in the laboratories, e.g., in ref. ^7,8^, in addition to the interactions occurring in the above-mentioned natural plasmas, e.g., interaction with the magnetic fields^9–11^. The electric field perpendicular to the physical boundary is spontaneously formed in the sheath and accelerates the ions toward the boundary; resulting in the transfer of the momentum flux perpendicular to the surface. However, if they have velocity components parallel to the boundary surface, they can also deliver their momentum parallel to the boundary. Therefore, it is important to identify both the momentum components perpendicular and parallel to the boundary for understanding the plasma dynamics in laboratories, although the latter one is often neglected in models^12–14^. Such momentum transfer and loss to the wall are indeed associated with various terrestrial plasmas ranging from the industrial to fusion plasmas. For example, the momentum transfer to the target plate in sputtering devices affect the material ejection from the target surface^15^. Even in magnetically-confined fusion plasmas, the plasma contacts to the physical boundary at a divertor, where the magnetic field lines intersect the physical wall and high heat flux flows to the divertor plate^16^. Therefore, the inhibition of the heat flux to the divertor is a challenging issue for fusion plasmas and the fundamental researches have been carried out^17^.

**Figure 1:**
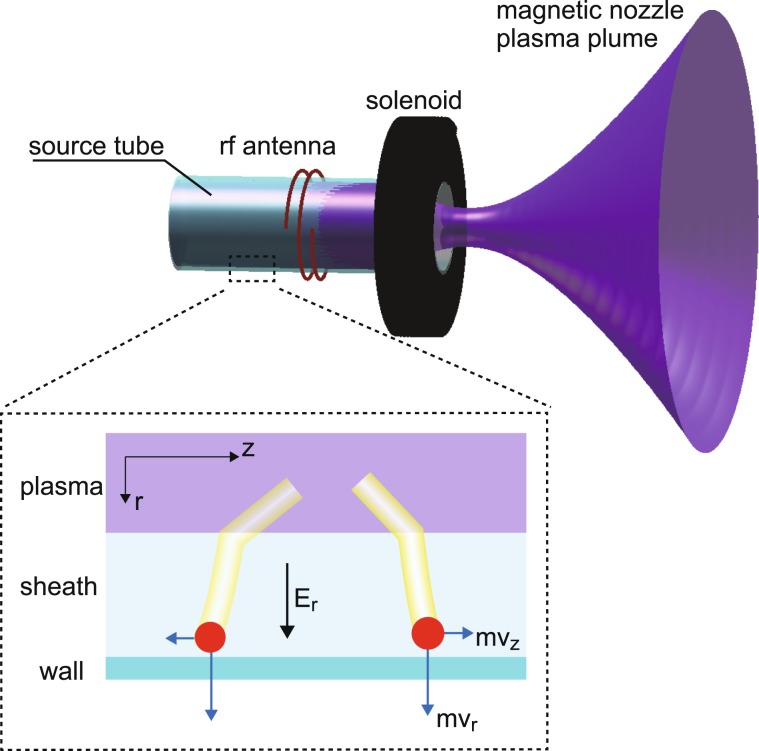
Magnetic nozzle rf plasma thruster configuration and physical description of the ions lost to the wall.

One of the fascinating applications of the plasma momentum flux is an electric propulsion device in space since the momentum flux is directly associated with the performance of electric propulsion devices^18^. The thrust force propelling a spacecraft is equivalent to the momentum flux exhausted from the system, where the reaction force is exerted on somewhere and somehow, e.g., to the acceleration grids by the electrostatic force in an ion gridded thruster^19^, to the physical axial wall by the sheath-accelerated ions^20–22^, and to the magnetic field by the Lorentz force^23–28^. Figure 1 shows the schematic diagram of a new type of the electric propulsion device called a magnetic nozzle radiofrequency (rf) plasma thruster^29,30^, which consists of an insulator source tube wound by an rf antenna and a solenoid providing a static magnetic field. Since the magnetic field eventually diverges downstream of the source tube, the magnetic nozzle is inevitably formed there. The plasma beam is spontaneously accelerated in the magnetic nozzle via various acceleration processes, resulting in the enhancement of the thrust. One of the experiments relating to the magnetic nozzle rf plasma thruster have implied that non-negligible axial momentum flux is lost to the radial source wall of a high density helicon source^31^, which has been discovered by the direct measurement of the total axial force imparted to the cylindrical source tube, while it has been assumed to be negligible in previous theories for simplification^12–14^. As already-mentioned, the ions lost to the wall will have significant radial momentum flux due to the ion acceleration in the sheath, where the electron pressure is converted into the ion dynamic momentum via the electric field, while they can deliver their axial momentum to the wall if they have some axial velocity components as sketched in Fig. 1. It seems to imply that the information of the central plasma is somewhat mirrored to the momentum loss process at the radial source boundary. Subsequent particle-in-cell simulation has analyzed the spatial profile of the momentum loss to the wall^32^, where the scale of the thruster has to be reduced because of the calculation time; the quantitative comparison between the simulation and the experiment has not been performed. Furthermore, the axial momentum flux lost to the radial wall would be directly connected with the recently proposed new concept of the space debris removal method with the magnetic nozzle rf plasma thruster^33^, since it does not contain the axial boundary but the thrust force has been detected. It is expected that the axial forces are exerted to the radial wall and the magnetic fields. However the detailed force components are still unclear. Hence, the direct, spatially-resolved, and vector-resolved measurement of the local momentum flux lost to the wall is required to understand how and where the plasma momentum flux is transferred; understanding the fundamental process in details will give insight into the improvement of the thruster performance.

Here the spatially- and vector-resolved measurement of the momentum flux near the radial source wall is performed by using a momentum vector measurement instrument (MVMI) described in ‘Method’ section, which is mounted on an axially movable stage immersed in vacuum and consists of a detector plate facing the radial center. As the detector plate is attached to the arm rotated by the radial force and this structure is further mounted on the axially movable pendulum, both the radial and axial force components exerted to the detector plate, i.e., the radial and axial momentum fluxes to the radially facing boundary, can be measured. The first, direct, spatially-resolved, and vector-resolved measurement of the momentum flux to the detector located near the radial source wall shows that both the radial and axial momentum fluxes lost to the radial wall are reduced by the magnetic field strength, in spite of the unchanged ion flux taken by a Langmuir probe. The energy loss to the wall is also assessed from the measured momentum fluxes and the ion current; implying the reduction of the energy flux to the wall by the magnetic field. These data are significantly important to understand the performance degradation mechanisms and to lead to a high performance plasma thruster.

## Results

**Figure 2:**
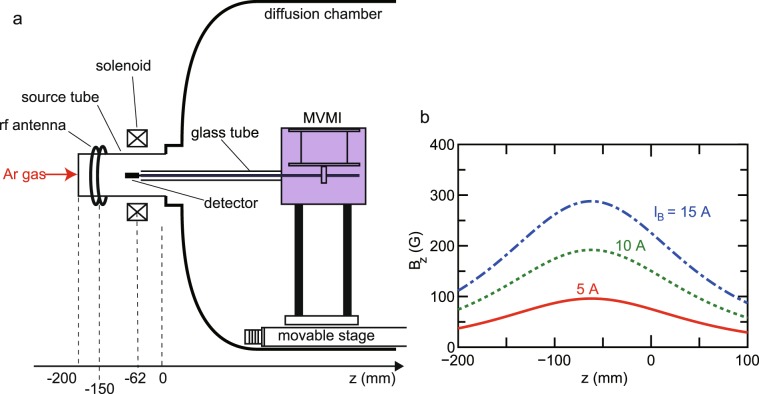
(**a**) Schematic diagram of the magnetic nozzle rf plasma thruster attached to the diffusion chamber. The MVMI is mounted on the axially movable motor stage and the detector plate attached to the MVMI arm is inserted into the source tube. (**b**) Calculated magnetic field strength on axis for the solenoid current of *I*_*B*_ = 5 A (a solid line), 10 A (a dotted line), and 15 A (a dotted-dashed line). This configuration enables to investigate the loss of the radial and axial momentum fluxes to the radial wall.

The experiment is carried out with the magnetic nozzle rf plasma thruster configuration [Fig. 2(a)]. The plasma source consisting of a 95-mm-inner-diameter and 200-mm-long pyrex glass source tube, a double-turn rf loop antenna, and a solenoid providing the static magnetic field, is attached to a 600-mm-diameter and 1400-mm-long vacuum chamber evacuated by a turbomolecular pumping system to a base pressure of an order of 10^−4^ Pa. The rf antenna is wound around the source tube at *z* = −150 mm and the solenoid is centered at *z* = −62 mm with the definition of *z* = 0 as the open source exit. Argon gas is continuously introduced from the upstream flange via a mass flow controller and the gas flow rate is maintained at 20 sccm resulting in a chamber pressure of about 80 mPa. A static magnetic field is applied by supplying a dc current *I*_*B*_ to the solenoid, where calculated profiles of the magnetic field strength on axis for various solenoid currents are drawn in Fig. 2(b). The magnetic field lines converge at the solenoid center and expand in the diffusion chamber, where some of the outer field lines intersect the radial source wall near the thruster exit. The rf antenna is powered by a 13.56 MHz rf generator via an impedance matching circuit. The rf power is chosen as 400 W and the matching circuit is tuned so as to minimize the rf power reflection; typical reflected power is less than a Watt. The momentum vector measurement instrument (MVMI) similar to the previous bench test^34^, which is described in ‘Method’ section, is mounted on an axially movable motor stage immersed in vacuum and a 20-mm by 30-mm detector plate facing the radial center is inserted into the source tube. The detector plate is displaced in the radial and axial directions by the radial and axial forces exerted to the detector surface, respectively. Both the displacements are measured by two different light-emitting-diode (LED) displacement sensors installed in the MVMI.

**Figure 3:**
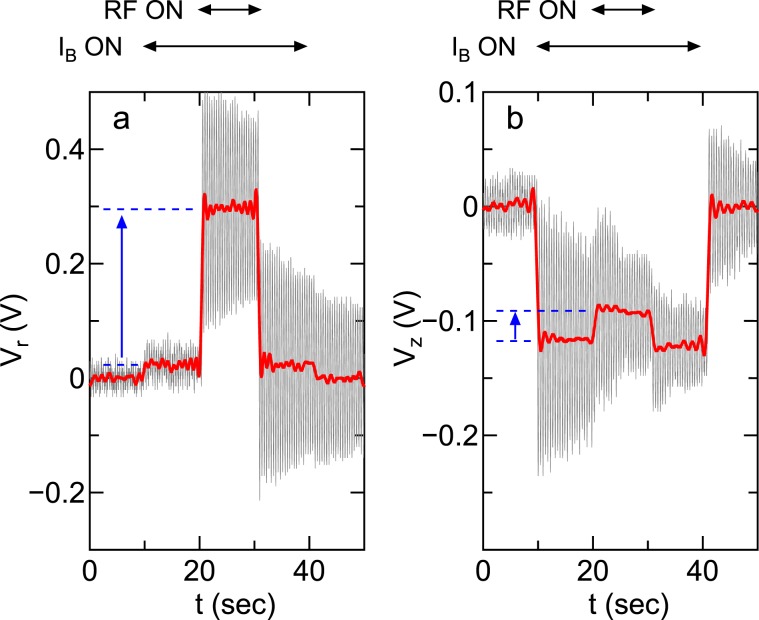
Raw (thin lines) and filtered (bold lines) signals from the (**a**) radial and (**b**) axial displacement sensors for the solenoid current of *I*_*B*_ = 5 A and the rf power of *P*_*rf*_ = 400 W, where the detector plate is located at (*z*, *r*) = (−50 mm, 40 mm). The displacement induced by the plasma can be estimated from the difference in the equilibrium positions between the ‘RF on’ and ‘RF off’ periods with ‘*I*_*B*_ on’ as indicated by the solid arrows and the dotted lines. The signals show that the radially outward and axially downward force is exerted to the detector plate.

Typical signals of the radial (*V*_*r*_) and axial (*V*_*z*_) displacement sensors of the MVMI are drawn by thin lines in Fig. 3(a,b), respectively. The solenoid current and the rf power are turned on for *t* ~ 10–40 sec and *t* ~ 20–30 sec, respectively. Since both the signals of *V*_*r*_ and *V*_*z*_ include the pendulum oscillation components with frequencies of about 2 Hz, the oscillating components are removed by performing the Fourier transform, the amplitude filter, and the inverse Fourier transform, as drawn by the bold lines. The detailed procedure of the signal analysis is described in ‘Method’ section. The positive displacements in the radial and axial sensors indicate that the radially outward and axially downward forces are exerted to the detector plate, respectively. Since the displacements are induced by only supplying the solenoid current due to the magnetic force on somewhere, e.g., stainless steel metallic parts and permanent magnets on the MVMI, the displacements induced by the plasma are estimated from the difference in the equilibrium signals between the ‘RF on’ and ‘RF off’ periods with ‘*I*_*B*_ on’ as indicated by the arrows and dotted lines in Fig. 3. The results in Fig. 3 demonstrate the vector-resolved measurement of the force, implying that the radially outward and axially downward momentum fluxes are lost to the radial wall.

**Figure 4:**
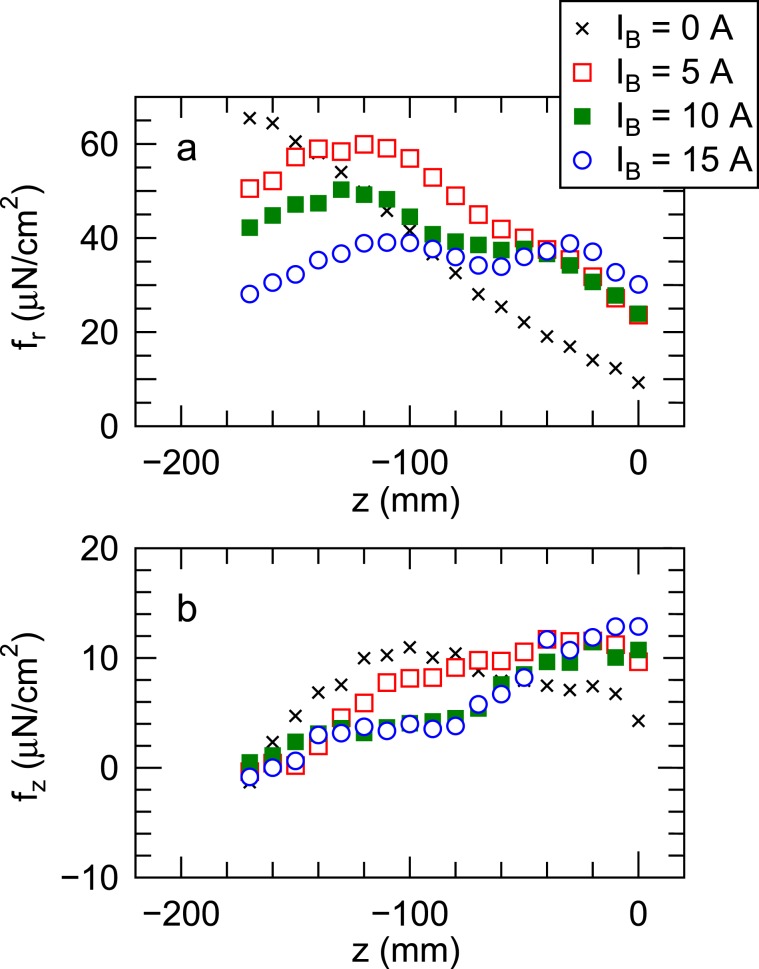
Axial profiles of the (**a**) radial and (**b**) axial force densities *f*_*r*_ and *f*_*z*_ for the various values of the solenoid current *I*_*B*_ and the constant rf power of *P*_*rf*_ = 400 W, where the positive values of *f*_*r*_ and *f*_*z*_ correspond to the radially outward and axially downward forces exerted to the detector plate. The uncertainty of the force density measurement is about ±5% and the resolution is less than 2 *μ*N/cm^2^. These are the direct identification of the profiles of the radial and axial momentum fluxes to the wall. The results show that the radial momentum flux lost to the radial wall is significantly reduced by the magnetic field strength. Furthermore, the axial momentum flux transferred to the wall is also reduced by the magnetic field strength.

The vector-resolved measurement calibrated with a coefficient relating the displacements to the forces is then performed as a function of the axial position *z* of the detector plate, where the radial position is maintained at *r* = 40 mm and the detailed calibration results can be found in ‘Methods’ section. Figure 4 shows the axial profiles of the radial (*f*_*r*_) and axial (*f*_*z*_) force densities for various values of the solenoid current *I*_*B*_. The radial force density *f*_*r*_ at *z* < −60 mm is found to decrease with an increase in the solenoid current, i.e., the magnetic field strength, as seen in Fig. 4(a). The downward axial force density also decreases around *z* ~ −100 mm when increasing the solenoid current, which seems to be consistent with the previous measurement of the axial force integrated over the inner surface of the source tube^31^. Both the *f*_*r*_ and *f*_*z*_ at *z* > −60 mm are unchanged here; it is probably due to the presence of the divergent magnetic field lines intersecting the radial wall there, along which the upstream plasma is transported. The data in Fig. 4 are the first experimental identification of both the radial and axial momentum fluxes lost to the wall and identify where the plasma momentum components are lost. The momentum transport and loss in the source are expected to be related with various physical processes arising from the magnetic field and geometric configurations^35^, the plasma-neutral interaction^12,36,37^, the non-Maxwellian energy distributions of ions and electrons^38–40^, and the plasma structural formations^41–44^. Hence more detailed investigation on the momentum transport physics still remains further challenge. It has to be mentioned that the rf power absorbed by the plasma is not constant when changing the magnetic field strength as shown later. The energy lost to the wall originating from the plasma loss to the wall will be discussed.

**Figure 5:**
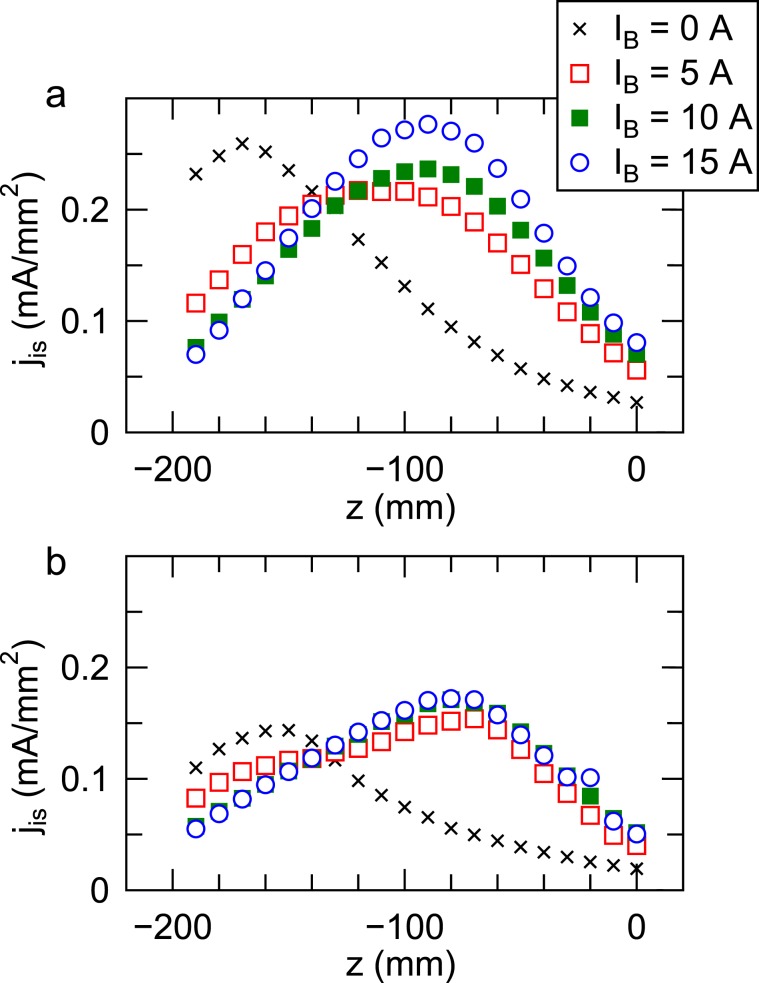
Axial profiles of the ion saturation current density *j*_*is*_ of the Langmuir probe at the radial positions of (**a**) *r* = 0 and (**b**) *r* = 40 mm. The change of the profile by applying the magnetic field can be clearly seen between the *I*_*B*_ = 0 case and the other cases, while no significant change is observed when increasing the solenoid current from *I*_*B*_ = 5 A to 15 A, implying that the ion flux to the radial source wall is not reduced by the magnetic field strength being tested here.

Axial profiles of the ion saturation current density *j*_*is*_ are measured by using a Langmuir probe (see ‘Method’ section) at *r* = 0 and *r* = 40 mm as plotted in Fig. 5(a,b), respectively. A clear change between the no magnetic field case (*I*_*B*_ = 0) and the other cases with *I*_*B*_ = 5, 10, and 15 A, can be seen. When comparing the data for *I*_*B*_ = 5 A, 10 A, and 15 A in Fig. 5(a), the maximum density position is found to shift toward the downstream side with the increase in *I*_*B*_. As the maximum density position generally has the maximum potential, the ions are accelerated axially and their axial momentum flux is increased via the electrostatic acceleration. When the axially accelerated ions are lost to the radial wall, they deliver their axial momentum to the wall, corresponding to the detected *f*_*z*_. The maximum density position is around *z* = −90 mm for *I*_*B*_ = 10 A and 15 A; *f*_*z*_ around *z* = −100 mm is considered to be reduced since only the radially accelerated ions can come from the maximum density position for these cases. The maximum loss of the radial momentum flux near the rf antenna (*z* = −150 mm) for the *I*_*B*_ = 0 case in Fig. 4(a) is consistent with the measured ion saturation current in Fig. 5(a), where the plasma produced near the rf antenna isotropically expands; the large momentum flux loss appears around the maximum ion saturation current position. No significant change of the ion saturation current corresponding to the ion flux can be seen near the wall when increasing the solenoid current from 5 A to 15 A as in Fig. 5(b); nevertheless the radial momentum loss is significantly reduced by the magnetic field as already shown in Fig. 4(a). These imply that the magnetic field can inhibit the radial acceleration of the ions at the wall sheath and the resultant loss of the radial momentum flux. The potential drop *ϕ*_*w*_ at the wall sheath is known to be proportional to the electron temperature, being *ϕ*_*w*_ ~ 4.7*k*_*B*_*Te*/e for a free fall model in argon^6^. The electrons are likely to be cooled along the radial axis by filtering the high energy electrons, being similar to the magnetic filter configuration^45,46^ and induces the decrease in the potential drop of the wall sheath. The other scenario is based on the balance of the electron and ion fluxes. According to the model by Chodura^47^, the magnitude of the potential drop *ϕ*_*w*_ decreases with an increase in *Γ*_*i*_/*Γ*_*e*_, where *Γ*_*i*_ and *Γ*_*e*_ are the fluxes of the ions and the electrons, respectively. Very qualitatively, both the diffusion coefficient and the mobility across the magnetic field are functions of ${(1+{\omega }_{c}^{2}{\tau }^{2})}^{-1}$, where *ω*_*c*_ and *τ* are the gyro-frequency and the collisional time, respectively. When considering the elastic collisions with the neutrals in the given pressure of 80 mPa and the typical magnetic field strength of 200 G, *ω*_*c*_*τ* for electrons and ions are orders of 10^3^ and 10^0^, respectively, where the collisional cross sections of 10^−19^m^2^ for *e-n* collisions and 4 × 10^−19^m^2^ for *i-n* collisions are used for the calculation. Therefore, the presence of the magnetic field can significantly inhibit the cross-field diffusion of the electrons rather than the ions, yielding the enhancement of *Γ*_*i*_/*Γ*_*e*_ and the reduction of the magnitude of *ϕ*_*w*_. It is considered that the change in *ϕ*_*w*_ due to the above-mentioned effects of the electron temperature and the fluxes dominates the loss process of the radial momentum flux to the wall.

Furthermore the energy flux lost to the wall is discussed here in addition to the directly measured radial and axial momentum fluxes. Let us start with assuming no magnetic field case. The radial and axial momentum fluxes to the radially facing detector plate, corresponding to the force densities *f*_*r*_ and *f*_*z*_, are generally given by

1 $${f}_{r}={m}_{i}{n}_{s}{u}_{rs}{u}_{rw}+{n}_{ew}{k}_{B}{T}_{e},$$

2 $${f}_{z}={m}_{i}{n}_{s}{u}_{rs}{u}_{zw},$$

where *m*_*i*_, *n*_*s*_, *u*_*rs*_, *u*_*rw*_, and *u*_*zw*_ are the ion mass, the ion density at the sheath edge, and the radial ion velocity at the sheath edge, the radial ion velocity at the wall, and the axial ion velocity at the wall, respectively. The second term of the right-hand side of Eq. (1) is the electron pressure given by the electron density *n*_*ew*_ at the wall, the Boltzmann constant *k*_*B*_, and the electron temperature *T*_*e*_. Since the electron density *n*_*ew*_ at the wall can be written as

3 $${n}_{ew}={n}_{s}\exp \left(-\frac{e{\phi }_{w}}{{k}_{B}{T}_{e}}\right),$$

with the voltage *ϕ*_*w*_ at the wall sheath and *ϕ*_*w*_ ~ 4.7*k*_*B*_*T*_*e*_ for argon^6^, the electron pressure in Eq. (1) is negligible, being about 1 % of *f*_*r*_. When applying the magnetic field parallel to the wall surface, the electron pressure term will be further reduced; hence *f*_*r*_ and *f*_*z*_ are given by

4 $${f}_{r}={m}_{i}{n}_{s}{u}_{rs}{u}_{rw},$$

and by Eq. (2), respectively, for both the cases.

Contrary to the momentum flux, the energy flux taken away by the electrons is non-negligible for the case of no magnetic field. Assuming an isotropic Maxwellian electrons, the radial electron energy flux *p*_*we*_ to the wall is given by^6^

5 $${p}_{we}=2{k}_{B}{T}_{e}{{\Gamma }}_{e},$$

where *Γ*_*e*_ is the electron flux to the wall and has to be balanced with the ion flux *n*_*s*_*u*_*rs*_ at the dielectric wall, i.e.,

6 $${{\Gamma }}_{e}={n}_{s}{u}_{rs}.$$

According to the Bohm criteria, the ions at the sheath edge have to have a velocity accelerated by a presheath voltage of *k*_*B*_*T*_*e*_/2*e*; the ions are totally accelerated by the voltage of 5.2*k*_*B*_*T*_*e*_/*e* due to the presheath and the sheath. Therefore the relation of

7 $$\frac{1}{2}{m}_{i}{u}_{rw}^{2}=5.2{k}_{B}{T}_{e},$$

can be obtained. The total energy flux *p*_*w*_ delivered by the ions (having finite *u*_*rw*_ and *u*_*zw*_) and the isotropic electrons is

8 $${p}_{w}=\frac{1}{2}{m}_{i}({u}_{rw}^{2}+{u}_{zw}^{2}){n}_{s}{u}_{rs}+{p}_{we}.$$

By combining Eq. (2), Eqs. (4–8), and the ion saturation current density of *j*_*is*_ = *en*_*s*_*u*_*rs*_, the energy flux to the detector plate is rewritten as

9 $${p}_{w}=\frac{e}{2{m}_{i}}\frac{(1+2/5.2){f}_{r}^{2}+{f}_{z}^{2}}{{j}_{is}}.$$

For the case that a sufficiently strong magnetic field parallel to the detector plate is applied, the energy loss by the electrons would be negligible; then

10 $${p}_{w}=\frac{e}{2{m}_{i}}\frac{{f}_{r}^{2}+{f}_{z}^{2}}{{j}_{is}}.$$

Eqs. (9) and (10) correspond to the maximum and minimum cases in the estimation of the energy flux by using the measured *f*_*r*_, *f*_*z*_, and *j*_*is*_, since the experimental condition would fit the intermediate condition between the no and finite magnetic field strength. Therefore, the energy flux is assessed by using both Eqs. (9) and (10).

**Figure 6:**
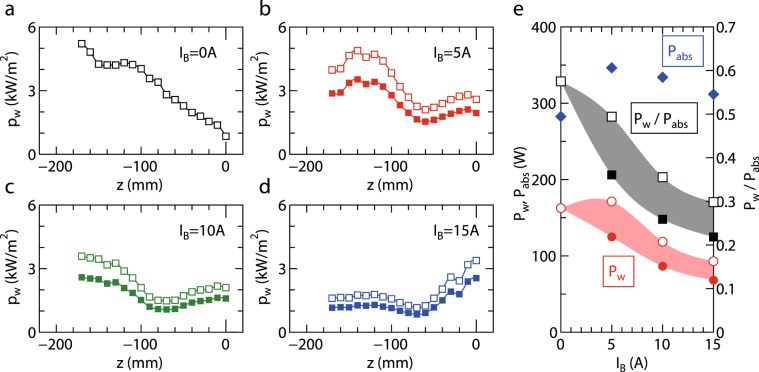
Axial profiles of the energy flux *p*_*w*_ lost to the radial wall for (**a**) *I*_*B*_ = 0 A, (**b**) 5 A, (**c**) 10 A, and (**d**) 15 A, where the open and filled squares are estimated from Eqs. (9) and (10), respectively. The solid lines are added as visual guides. (**e**) The total power *P*_*w*_ (open and filled circles), the rf power *P*_*abs*_ absorbed by the plasma (filled diamonds), and the normalized power *P*_*w*_/*P*_*abs*_ (open and filled squares) as functions of the solenoid current *I*_*B*_, where the open and filled symbols in *P*_*w*_ and *P*_*w*_/*P*_*abs*_ are from Eqs. (9) and (10), respectively. Since the open and filled symbols in Fig. 6(e) correspond to the upper and lower limits of the estimation, the actual power loss is within the colored region. The results demonstrate that the energy loss to the wall is inhibited by the magnetic field strength, where the energy loss at the upstream side (*z* < −60 mm) is found to be mainly reduced.

By using the data in Figs. 4 and 5(b), the energy fluxes estimated from Eqs. (9) and (10) are plotted by open and filled squares, respectively, in Fig. 6 for (a) *I*_*B*_ = 0 A, (b) 5 A, (c) 10 A, and (d) 15 A cases. The energy flux lost to the radial wall is clearly reduced by increasing the magnetic field strength despite the unchanged ion flux observed in Fig. 5(b). Since the rf power transfer efficiency is often changed by the external magnetic field strength^48^, the energy lost to the wall should be normalized by the absorbed rf power to discuss the effect of the magnetic field strength on the energy loss rate. The rf power transfer efficiency *η*_*p*_ can be obtained by measuring the rf antenna current as described in ‘Methods’ section. The absorbed rf power *P*_*abs*_ obtained by multiplying *η*_*p*_ to the generator output power of 400 W is plotted by filled diamonds in Fig. 6(e). Assuming the axisymmetric profile, the total power *P*_*w*_ lost to the wall is obtained by integrating the data in Fig. 6(a–d) as plotted in Fig. 6(e), where the open and filled circles are from Eqs. (9) and (10), respectively, and Eq. (10) is not used for *I*_*B*_ = 0. Furthermore, the normalized power *P*_*w*_/*P*_*abs*_ based on Eqs. (9) and (10) are also plotted by open and filled squares, respectively. Since these equations correspond to the upper and lower limits of the power estimation, the actual loss power and normalized power would be in the colored region in Fig. 6(e). These data demonstrate that the energy lost to the radial wall can be inhibited by the magnetic field strength even in the weakly magnetized (magnetized electrons and unmagnetized ions) condition. Based on the measured *f*_*r*_ larger than *f*_*z*_, the radially accelerated ions seem to be responsible for the energy loss to the wall, rather than the axially accelerated ions.

It should be noted that the plasma source is operated over the magnetic field ranging from 0 G to about 300 G, where the rf power coupling is changed by the magnetic field strength. It is considered that the inductively coupled and helicon wave coupled modes are superimposed, depending on the operation parameters. Although the rf power is coupled with the electrons^49^, the power absorption and electron heating profiles would be changed by the excitation of the helicon wave. Further investigation on the rf electromagnetic field will be required to fully understand the energy and momentum transport dynamics in the magnetic nozzle rf plasma thruster.

## Conclusion

The spatial profiles of the radial and axial momentum fluxes lost to the radial wall of the magnetic nozzle rf plasma thruster are measured by using the momentum vector measurement instrument. The first, direct, individual, and simultaneous identifications of the radial and axial momentum fluxes demonstrate that the radial momentum flux to the plasma source wall is significantly reduced by increasing the magnetic field strength, resulting in the inhibition of the energy loss to the wall. Simultaneously, the axial momentum lost to the radial wall is also reduced by the magnetic field strength, which is considered to be due to the change of the plasma profile and the resultant changes in the location of the ion acceleration and the plasma transport to the wall. These measurements are significantly useful to understand how and where the plasma momentum and the energy are lost in the thruster and further applicable to the other terrestrial plasmas.

## Methods

### Momentum vector measurement instruments

**Figure 7:**
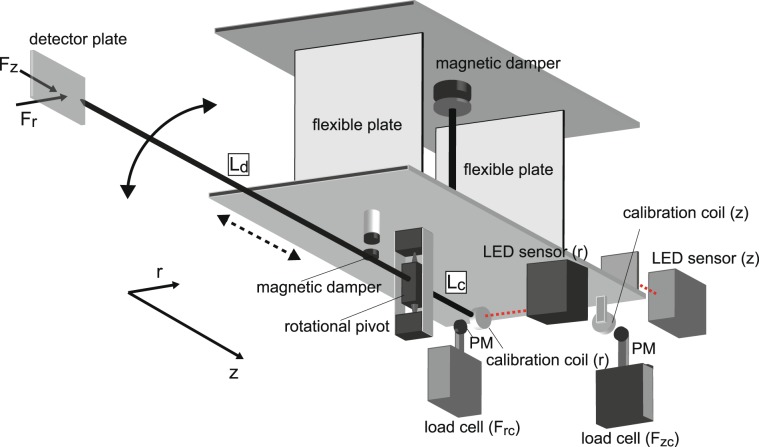
Schematic diagram of the momentum vector measurement instrument (MVMI).

Figure 7 shows the detailed structure of the momentum vector measurement instrument (MVMI), which is very similar to the previous bench test^34^ and the preliminarily test in plasmas^50^. The whole structure of the MVMI is flipped from the previous configuration to stabilize the equilibrium position of the axial pendulum when moving the whole structure by the motor stage. The momentum detector plate of 20 mm in height and 30 mm in width is attached to an arm supported by a rotational pivot, which is further mounted on an axially movable pendulum consisting of two flexible plates. The surface of the detector plate is directed to the radially inward direction and exposed to the plasmas, while the back side of the plate and the arm are covered by insulator structure, whereby the momentum transfer to the structures except the detector surface is minimized. Furthermore, the structure of the dual pendulums is covered by metallic parts for shielding it from the rf noise and the plasma. When the ions having the radial and axial momentums impinge the detector surface, both the radial and axial forces are exerted to the detector and transferred to the rotational and axially movable balances, respectively. By measuring the displacements in the radial and axial directions simultaneously by the two LED displacement sensors, both the radial and axial force components can be obtained by multiplying the calibration coefficients relating the displacements to the forces.

**Figure 8:**
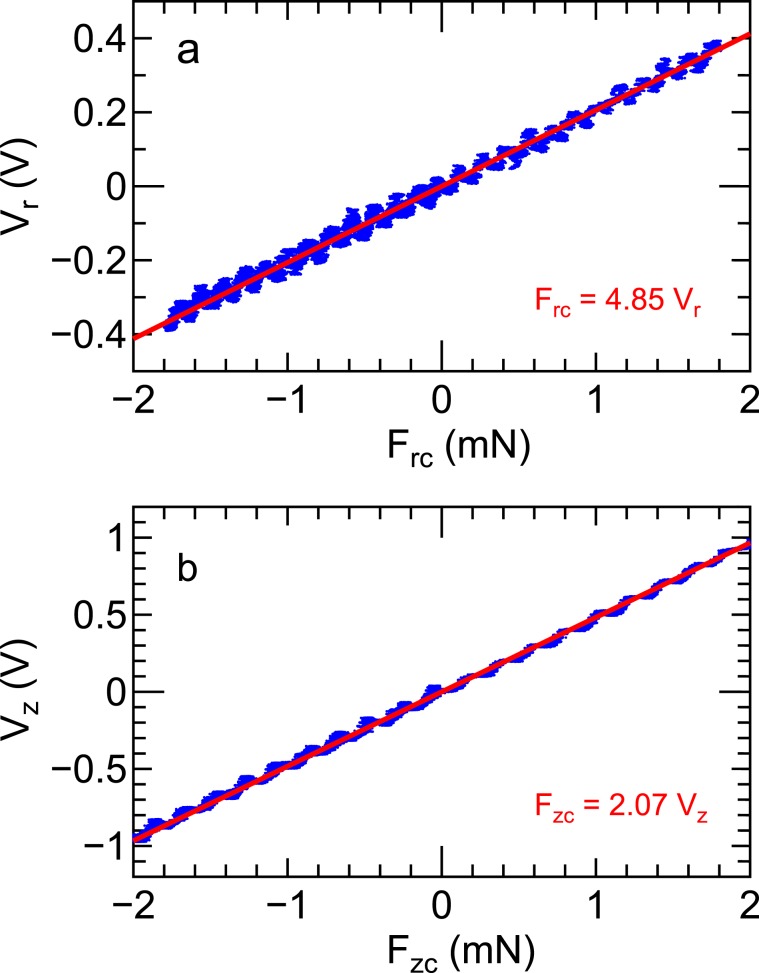
The forces (*F*_*rc*_, *F*_*zc*_) measured by the load cells and the displacement sensor signals (*V*_*r*_, *V*_*z*_) (dots) when slowly sweeping the current of the (**a**) radial and (**b**) axial calibration coils. The linear fitted lines giving the calibration coefficients shown by the inset texts in Fig. 8 are drawn by the bold lines.

The calibration coefficient can be obtained by measuring the displacement and the force simultaneously in each direction when sweeping the force exerted to the structure. Two calibration coils directed in the radial and axial directions are attached to the arm and the axially movable pendulum; load cells including the permanent magnets (PMs) facing the calibration coils are located as drawn in Fig. 7. By sweeping the calibration coil current and measuring the displacement by the LED sensor and the force by the load cell, the calibration coefficient can be obtained for each direction as demonstrated previously^51^. Figure 8 shows the measured relations between the force and the displacement sensor signal in the (a) radial (*V*_*r*_-*F*_*rc*_) and (b) axial (*V*_*z*_-*F*_*zc*_) directions, where the signals include some oscillating components of the pendulum. It is noted that the cross talk between the two directions has been negligible in the previous bench test and is also confirmed to be negligible in the present experiment. The obtained data are fitted by linear lines as plotted by the bold lines and as shown by the inset texts in Fig. 8. Since the radial displacement is actually related with a torque rather than the force, the radial force to the detector plate *F*_*r*_ can be related with the force applied to the radial calibration coil *F*_*rc*_ by multiplying *L*_*c*_/*L*_*d*_ to the coefficient in Fig. 8(a), where *L*_*c*_(= 70 mm) and *L*_*d*_(= 375 mm) are the distances from the pivot to the calibration coil and to the detector plate, respectively. Therefore, the absolute values of the radial and axial forces exerted on the detector plate can be obtained as

11 $${F}_{r}=\frac{{L}_{c}}{{L}_{d}}{F}_{rc}\simeq 0.91{V}_{r},$$

12 $${F}_{z}={F}_{zc}=2.07{V}_{z},$$

respectively. Since the estimated resolution of the force is about 10 *μ*N^34^ and the detector plate has the surface area of 6 cm^2^, the force density resolution is less than 2 *μ*N/cm^2^ for both the directions.

### Displacement signal analysis

**Figure 9:**
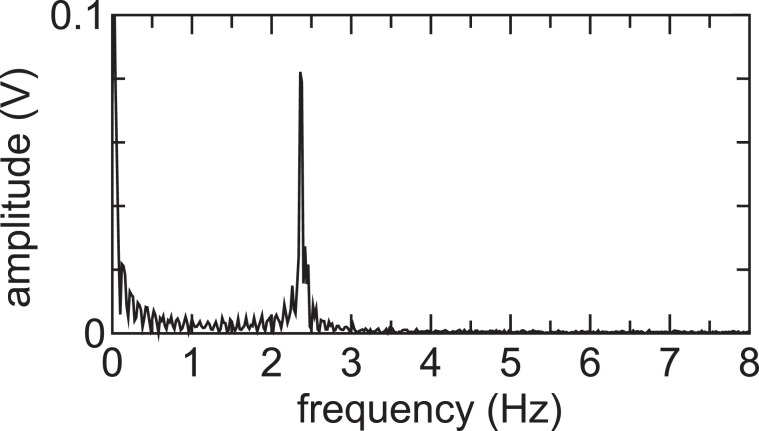
Amplitude spectrum of the displacement signal plotted by the thin line in Fig. 3(a).

The displacement signal by the MVMI includes the oscillating component inherent to the pendulum motion. A frequency spectrum of the raw data [thin line in Fig. 3(a)] can be obtained by Fast Fourier Transform (FFT) and the amplitude spectrum is shown in Fig. 9. It is found that the coherent oscillating component around 2-3 Hz is found to exist. After multiplying a filter function, which has the gain of unity at *f* < 1 Hz and zero at *f* ≥1 Hz, to the frequency spectrum, it is converted into the temporal signal via Inverse Fast Fourier Transform (IFFT); the filtered signal as shown by the bold line in Fig. 3(a) can be obtained.

### Langmuir probe

The Langmuir probe used in the present experiment has a 3-mm-diameter planar tip facing the radial center, where the opposite surface is covered by a ceramic past. The probe is biased to −70 V through a resistor and the ion current can be obtained from the voltage across the resistor. Since the ion Larmor radius is a few of cm even for the maximum magnetic field and larger than the probe tip size, the presence of the magnetic field does not affect the ion current measurement. By mounting the probe on the axially and radially movable stage, the axial profiles of the ion current at the different radial positions can be obtained as shown in Fig. 5.

### rf power transfer efficiency

The rf power transfer efficiency can be given by the ratio of the power absorbed by the plasma to the total rf power as seen in many literatures, e.g., ref. ^48^. Assuming that the rf power is consumed by the plasma and the rf antenna, the power transfer efficiency *η*_*p*_ can be written as

13 $${\eta }_{p}=\frac{{R}_{p}}{{R}_{total}}=\frac{{R}_{total}-{R}_{ant}}{{R}_{total}},$$

where *R*_*p*_, *R*_*ant*_, and *R*_*total*_ are the resistances of the plasma, the rf antenna, and the total load (including the antenna and the plasma) during the discharge. *R*_*total*_ and *R*_*ant*_ are estimated from the measured rf antenna current and the net rf power with the plasma, and with no plasma (i.e., no gas), respectively, where the impedpance matching is tuned so as to minimize the power reflection (typically less than a Watt) for all the cases with and without the plasma.
